# Analysis of the National Institutes of Health COVID-19 Neuro Databank by geographic region and income status

**DOI:** 10.21203/rs.3.rs-7371770/v1

**Published:** 2025-10-06

**Authors:** Jacqueline C. Dominguez, Krizelle Cleo Fowler, Igor J. Koralnik, Kore Liow, Encarnita Ampil, Qi Zhi, Lina Laxamana, Remy Berroya, Cecile J. Noris, Sharon B. Meropol, Andrea B. Troxel

**Affiliations:** St. Luke’s Medical Center; Institute for Dementia Care Asia; Northwestern Feinberg School of Medicine; Hawaii Pacific Neuroscience; St. Luke’s Medical Center; Hawaii Pacific Neuroscience; St. Luke’s Medical Center; St. Luke’s Medical Center; NYU Grossman School of Medicine; NYU Grossman School of Medicine; NYU Grossman School of Medicine

**Keywords:** Neuro COVID-19, Long COVID-19, Comparative analysis

## Abstract

**Background:**

While COVID-19 is more commonly known to present and persist in terms of respiratory symptoms, evidence for neurologic manifestations is limited.

**Objectives:**

This study aims to provide an epidemiological overview of neurologic manifestations of COVID-19. It compares the demographic and clinical profiles of patients based on geographic location and country income classification. Moreover, it describes the neurologic manifestations of Long COVID.

**Methods:**

This was a cross-sectional analysis of a multi-country cohort of patients with COVID-19-associated neurologic symptoms enrolled in the COVID-19 Neuro Databank from January 2020 to February 2025. Demographic, clinical and health system-related factors were described. Comparisons among geographic regions and income classifications were performed via analysis of variance and chi-square tests or Fisher’s exact tests.

**Results:**

Majority of the 3,901 patients were from the United States (U.S.) and from high-income countries. The mean age was 57.27±18 years and 54% were males. Neurologic comorbidities were highest in the U.S. whereas non-neurologic comorbidities. Asia had the greatest frequency of severe COVID and mortality. Vaccination and use of COVID-19 medication was lowest in Africa. There were significant associations between COVID-19 vaccination and use of COVID-19 medications with income level. The five leading COVID-19-associated neurologic conditions were neurocognitive disorders, fatigue, headache, anosmia, and ageusia. A subset of Long COVID was identified as thought disorders, fatigue, mood disorders, stroke, headache, and neurocognitive disorders.

**Conclusions:**

This study provides insight into the varying profile and burdens of COVID-19 associated neurologic conditions and the health inequities during the pandemic among geographic and income groups.

## 1. Introduction

Coronavirus disease 2019 (COVID-19) has had a catastrophic global impact since its appearance in 2020, and its effects persist despite the resolution of the acute phase of the pandemic. While respiratory involvement is most common, neurologic symptoms (Neuro-COVID) resulting from direct viral invasion, a hyperinflammatory state, cytokine storm, and cerebrovascular disease are prevalent [1, 2]. Neurologic symptoms may persist long after acute infection and decrease quality of life [3, 4]. Neuro-COVID disproportionately affects younger adults, potentially hindering productivity and increasing the healthcare burden [3, 5]. Much needs to be understood about Neuro-COVID and its global burden, especially with respect to health inequity, which became prominently apparent during the pandemic [6].

Few large multi-country cohorts have been established to study Neuro-COVID. The Global Consortium Study of Neurologic Dysfunction in COVID-19 (GCS-NeuroCOVID) reported the incidence and outcomes of patients enrolled from the United States and Africa, whereas the European Academy of Neurology (EAN) Neuro-COVID Registry (ENERGY) includes data from Europe, Asia and Africa [1]. The COVID-19 Neuro Databank was created to gather data from a substantial global cohort of individuals from diverse populations in different regions who developed neurologic conditions related to COVID-19 [7]. It is hypothesized that sociocultural and economic differences among different regions may be associated with the neurologic manifestations and outcomes of COVID-19. In this paper, we describe population-level differences by geographic regions and income status of patients in the COVID-19 Neuro Databank in terms of demographic and clinical profiles, and health system-related characteristics. Additionally, we report the profile of new or worsening neurologic conditions that fit the criteria of Long COVID by lasting at least 3 months [3].

## 2. Methods

### 2.1 Data sources

The study population was derived from COVID-19 Neuro Databank (NeuroCOVID), a large cohort of adults with new or worsened neurologic conditions associated with COVID-19 illness. The databank was launched in January 2020 in the early phase of the pandemic and until June 2025. Initial sites were recruited in the United States (U.S.), and international sites were later added, focusing on diversity and populations with data not described in published literature to avoid the risk of duplication of data. The participating sites were asked to contribute to a REDCap database of deidentified patients’ data using standardized common data elements [7]. All patients enrolled in the databank until February 2025 were included in this report.

### 2.2 Neurologic conditions

Neurologic conditions were identified based on clinical criteria when available. COVID-19-related neurologic conditions were those that were temporally related to COVID-19. The number of days between the onset of COVID-19 illness and the first new or worsened neurologic condition was recorded. When there was a question about COVID-19 relatedness, verification was made with the primary physician or the P.I. COVID-19 related neurologic conditions included acute and post-acute conditions and their duration. They were reported as new or worsening of a pre-existing comorbid neurologic condition. The persistence of symptoms reported by the patient or documented persistent signs when the patient was last seen were recorded. Long COVID was based on the consensus definition issued by the National Academies of Science Engineering and Medicine of a condition that occurs after SARS-CoV-2 infection and has been present for at least 3 months, with varied symptoms that affect one or more organ systems. Patients with neurologic Long COVID diagnoses were included [3].

### 2.3 Study Conceptual Framework

This cross-sectional study described the regional differences in the profiles of adults with new or worsened neurologic conditions associated with COVID-19. The top neurologic conditions and those that lasted more than 3 months were explored. Patient populations were described in terms of sociodemographic, clinical, and health system factors across the different countries and income groups. Countries were grouped according to the world regions: Asia (Malaysia, Philippines), Africa (Egypt), Europe (Spain, Poland, Norway), and the United States. The income level groups were based on the World Bank classification as middle-income countries (MICs), which are composed of Malaysia, the Philippines, and Egypt, and high-income countries (HICs), which are composed of European countries and the United States.

### 2.4 Data management and analysis

Site-level data were merged to allow regional comparisons. The data in REDCap were reported as collected. No imputation was performed on missing data. Missing or unknown values were reported. There were no a priori sample size or power calculations. The total sample size available for each data element was reported. Descriptive statistics were used for demographic and clinical characteristics, health system factors, COVID-19-related neurologic manifestations, and neurologic Long COVID, and were reported for the whole cohort and for each group. Comparative analysis, using ANOVA or Chi-Square/Fisher’s Exact tests, of key variables against region groups was also performed. All analyses were conducted in Stata ver. 18. This project was approved by a single IRB oversight, project number s20–01277, administered by the Office of Science and Research, NYU Langone Health.

## 3. Results

### 3.1 Population grouping.

A total of 3,901 individuals were included in the study. Most of the patients (79.3%) were from the U.S., where most of the initial sites were located, and the rest were from Asia, Africa, or Europe. Majority belonged to HIC. (Table 1)

### 3.2 Demographic profile.

The mean age differed across region groups (p < 0.001), with those in Asia being the oldest (66.6 ± 14.7 years). (Table 2) The age ranged from 18 to 90 + years, but the majority were between 45 and 75 years old. The sample was balanced in terms of sex, except in Asia, where there was a slight male predominance (57.4% males and 42% females). There was no significant association between sex and region (p = 0.350). The mean age and sex did not differ significantly between income groups. (Table 3) The sample was mostly non-Hispanic. In terms of education, there were patients with unknown or missing data (84%).

### 3.3 Neurologic comorbidity.

More than half of the sample had a neurologic comorbidity, and this was highest in the U.S. The top five neurologic comorbidities were as follows: 1. Psychiatric conditions (mood or thought disorders, 24%), 2. Stroke (ischemic stroke, transient ischemic attack, intracerebral hemorrhage, subarachnoid hemorrhage, 15%), 3. Headache (11%); 4. Epilepsy (11%), and 5. Dementia and neurodegenerative disease (Parkinson’s disease, Alzheimer’s disease, 11%). There was a significant association between the presence of any neurologic comorbidity and region, with Europe and the U.S. reporting high frequencies at 48.4% and 61.1%, respectively, compared with 8.8% and 34.9%, respectively, for Africa and Asia (p < 0.001). The highest frequency of comorbid stroke and dementia/neurodegenerative disease was in Asia, and psychiatric conditions were highest in Europe. (Table 4)

### 3.4 Non-neurologic comorbidity.

Seventy-five percent of the sample had non-neurologic comorbidities. Asia recorded the highest frequency (91.4%), compared with 59.6%, 74.2%, and 77.5% for Africa, Europe, and the U.S., respectively. (Table 4) Vascular risks were common in all regions. There was a significant association between the presence of non-neurologic comorbidity with region and income groups (p < 0.001). HICs had significantly greater non-neurologic comorbidity. (Table 5) The top five non-neurologic comorbidities, the majority of which are vascular risk factors, were highest in Asia: 1. Hypertension, 2. Diabetes, 3. Hyperlipidemia, 4. Obesity, and 5. Asthma. There were significant associations of each of these non-neurologic comorbidities with region and income group. (Tables 4 and 5)

### 3.5 Severity of COVID illness.

One-third of the sample had mild or moderate COVID, 37.8% and 32.1%, respectively. Severe COVID was highest in Asia (24.9%) and lowest in the U.S. (13.0%). Mild COVID was highest in the U.S. and significantly greater in the HIC (< 0.001). (Tables 4 and 5)

### 3.6 Health system-related profile.

Insurance coverage was high, ranging from 97.8–100.0% of the samples in each region. There was a significant association between having insurance and region group. Fewer than half of the samples received COVID-19 immunization (44.4%). Africa had the lowest frequency of vaccination (21.0%), whereas Europe had the highest (63.5%). Africa also had the highest degree of unknown information regarding vaccination (29.6%). (Table 6) Receipt of COVID-19 medication was recorded in 58.7% of the patients in the whole sample; it was highest in Asia (96.2%), lowest in Africa (3.6%), and lowest in MICs. (Tables 6 and 7) There were significant associations between the use of COVID-19 medication and both geographic and income group. There were significant associations between COVID-19 vaccination and use of COVID-19 medications with income level. (Table 7)

Two-thirds of the patients were hospitalized (61.6%), of whom 64.3% were admitted to the intensive care unit (ICU). Hospitalization was significantly greater in Asia (95.7%) than in Africa, Europe and the U.S. (77.2%, 62.9% and 56.6%, respectively, p < 0.001). (Table 6) There were significant associations between income level and hospitalization and ICU admission (Table 7). Hospitalization was significantly greater in the MIC group, whereas ICU admission was greater in the HIC group. Despite having the fewest hospitalizations, ICU admission was highest in the U.S. at 72.5%, whereas it was lowest in Europe at 25.6%. The mean length of hospital stay was 15 days, and it was significantly longer in Asia at 23 days and shortest in Africa at 9 days. The mean duration of stay in the ICU was 13 days. Hospital and ICU stays were longest in Asia (p < 0.001). The mortality rate was 27.4% for the whole sample, was highest in Asia at 35.9%, and was significantly higher in the MIC (p < 0.001). For a considerable proportion of the sample (22.6%), information on whether the patient died was unknown.

### 3.7 Neurologic conditions associated with COVID-19.

New neurologic conditions were frequent in all regions, ranging from 68.2% in the U.S. to 98.3% in Africa. A small proportion of the population experienced worsening of pre-existing neurologic conditions. (Table 8) The five leading neurologic conditions were 1) neurocognitive disorders, 2) fatigue, 3) headache, 4) anosmia, and 5) ageusia. The Neuro-COVID profile was similar in the U.S. and Europe but varied in Asia and Africa. Neurocognitive disorders were the first and second leading conditions in the U.S. and Europe, respectively, whereas they were the fifth leading condition in Asia and not the leading condition in Africa. Fatigue consistently led in all regions and was first in Europe and Africa. Headache was reported by 28% of all patients but was not a leading condition in Europe. Anosmia and ageusia were reported by 22% of the patients but only led in the U.S. and Europe. While stroke was not in the top five conditions, it was the most common in Asia and the fourth most common in Africa. Mood disorders, thought disorders, and seizures have been reported only in Europe, Africa, and Asia, respectively. When analyzed by income group, new neurologic conditions were more common in HIC (93.2% vs. 68.4%). The HICs had the same top neurologic conditions as the whole sample did, whereas for the LMIC, only fatigue and headache were consistently the top conditions. Other conditions in this group included mood, stroke, and thought disorders. (Table 9)

### 3.8 Neurologic Long COVID.

Six neurologic conditions were identified from a subset of patients who were not in the acute phase of COVID-19 and with a duration of > 3 months: 1) thought disorders (23.4%), 2) fatigue (9.7%), 3) mood disorders (8.9%),4) stroke (5.5%),5) headache (1.9%) and 6) neurocognitive disorders (1.7%). Fatigue and headache were the Long COVID symptoms reported across all regions and income groups. More than 90% of fatigue, mood and thought disorders; 65.2% of neurocognitive disorders; and 64.7% of headache cases were new, whereas the majority (55.2%) of stroke cases were exacerbations of pre-existing cerebrovascular disease. (Table 10)

## Discussion

The results describe and compare data from a substantial cohort of individuals from different geographic regions and income groups in the COVID-19 Neuro Databank. To our knowledge, this is the first study to analyze and report on COVID-19-associated neurologic conditions from a regional and economic perspective at the population level. Such analysis by regional and income groups is relevant considering the reported disproportionate impact of the pandemic on certain population groups, particularly those affected by socioeconomic inequities. The data include underrepresented regions such as Asia and Africa, as well as low- and middle-income country populations, which are not commonly reported in other cohorts [7].

Majority of the patients were white, and the Hispanic ethnicity was minority. These data are distinct from those of the Researching COVID to Enhance Recovery (RECOVER) study with a larger Hispanic population [8]. Beyond the diverse sociodemographic profiles, the results highlight differences in COVID-19 severity, comorbidity risk profiles, and health systems across different regions and income groups. The results confirm that the COVID-19 Neuro Databank is a significant initiative that documents neurologic complications associated with COVID-19, providing a broad dataset that includes patients from various geographic regions and income groups, making it a valuable resource for understanding disparities in COVID-19-related neurologic conditions [9].

Because the COVID-19 NeuroDatabank includes patients infected at the beginning of the pandemic through August 2024, the dataset captures the neurologic impact of COVID-19 across different variants. Research has shown that the neurologic complications associated with COVID-19 vary depending on the virus variant, with some waves showing higher rates of cerebrovascular diseases and others with fewer smell and tastedisturbances [10]. The results also included both acute neurologic conditions and post-acute sequelae lasting for 3 months. The cohort therefore provides valuable opportunities to analyze how neurologic conditions evolved throughout the pandemic and across different populations.

Although almost all patients had health insurance coverage, therefore ensuring access to care and better outcomes, significant disparities remained in terms of vaccination rates, the use of COVID-19 medication, hospital admissions and duration, and mortality outcomes. Immunization was lower in MICs. This is despite the reported greater willingness in MICs to vaccinate against COVID-19 and the perception of vaccines as safe and effective [11]. In this study, education levels seem to have played a role, as lower levels of formal education in MICs were associated with lower rates of vaccination, and individuals may have had less access to reliable health information. Low level of education as well as misinformation could impact the decision to vaccinate [12, 13]. Europe had the highest vaccination rate (90.4%), and Africa had the lowest, probably because of reported vaccine nationalism and supply chain issues that led to limited access to vaccines, despite global efforts such as the COVID-19 Vaccines Global Access (COVAX) [14, 15]. The disparities across regions emphasize the importance of strong public health communication to address misinformation and implement equitable vaccine distribution.

COVID-19 medications were provided to majority of the patients in most regions. However, in Africa, despite 100% insurance coverage, only 3.6% of patients have received COVID-19 medications, likely due to the weakness of the healthcare infrastructure [14]. In contrast, the Philippines saw government subsidies through the Philippine Healthcare Insurance Corporation (PhilHealth) to cover medical expenses beyond standard coverage [16]. In spite of this, Asia recorded the highest mortality, possibly due to greater comorbidities, greater disease severity, and lower vaccination rates. These findings highlight the need for global action toward equitable healthcare policies that go beyond insurance coverage to address systemic challenges in healthcare delivery.

The spectrum of COVID-19-related neurologic conditions in this study has been previously reported [17]. However, we identified differences across income groups. Middle-income countries had a greater propensity to have new neurologic conditions and hospitalizations, probably due to a higher frequency of moderate-to-severe disease (62.6%) than HIC (39.4%). Severe COVID-19 is associated with neurologic conditions due to systemic inflammation and neuronal damage [17, 18]. The most frequently reported COVID-19-related neurologic conditions differed across regions and income levels. Neurocognitive disorders have not been reported in Africa, and stroke has been reported only in Asia and Africa. Low awareness of cognitive symptoms and the need for cognitive testing may have led to underreporting of neurocognitive disorders. Vascular risk factors were more common in Asia and Africa, which most likely contributed to the greater occurrence of stroke and was aggravated by the greater severity of COVID-19.

Although this was a cross-sectional study, the database was able to gather data from some patients whose symptoms persisted longer than 3 months at the time of enrollment in the database. The neurologic Long COVID conditions included thought disorders, fatigue, mood disorders, stroke, headache, and neurocognitive disorders. All the neurologic Long COVID cases, with the exception of stroke, were new occurrences. The results confirm previous reports that incident neurologic and psychiatric morbidity can persist long after a COVID-19 infection [19]. These long-term conditions could impair people’s quality of life, hamper productivity, and strain the healthcare system. These results raise awareness of the substantial contribution of Long COVID to the global burden of mental health, especially considering the millions infected by COVID-19.

The study acknowledges several limitations. The data included a mix of patients seen in either the acute phase of COVID-19 or in post-COVID-19 clinics. The population is biased toward the beginning of the pandemic, with a high rate of hospitalization for severe COVID-19. As a cross-sectional study, there is incomplete data about post-COVID-19 conditions. There is also a possibility of selection bias given the limited inclusion of healthcare facilities per country and overrepresentation of the U.S. population. Moreover, the study also considers the underlying differences between the countries included in this ecological analysis, which could affect the results and their transferability in other settings. Nonetheless, this study could still contribute to the growing evidence base on COVID-19 associated neurologic conditions.

## Conclusion

The characteristics of the population in the Neuro-COVID database differ across Asia, Africa, Europe, and the U.S. and between HICs and MICs. The profile of neurologic conditions associated with COVID-19 likewise differs. Future studies are needed to examine the impact of these differences to address global health inequities. The diversity of the population and the wide range of neurologic conditions, spanning the beginning of the pandemic up to 2024, make the COVID-19 Neuro Databank a rich resource for research on the neurologic impact of COVID-19 across different variants, severity levels, patient demographics, and health systems, with a focus on equitable access during a pandemic, irrespective of economic status.

## Supplementary Material

Supplementary Files

This is a list of supplementary files associated with this preprint. Click to download.

• NeuroCOVIDlistofcollaborators.xlsx

## Figures and Tables

**Table 1:** Profile of Study Participants

	Summary
N	3,901
Country Group	
Africa: Egypt	535 (13.7%)
Asia: Malaysia, Philippines	209 (5.4%)
Europe: Poland, Spain, Norway	62 (1.6%)
United States	3,095 (79.3%)
Income Group	
Middle Income	744 (19.1%)
High Income	3,157 (80.9%)

**Table 2:** Demographic Characteristics per Region

Characteristics	Region Group					p-value [a]
	Africa	Asia	Europe	US	Total	
N (%	535 (13.7%)	209 (5.4%)	62 (1.6%)	3,095 (79.3%)	3,901 (100.0%)	
age (Mean (SD)	54.493 (18.19)	66.627 (14.70)	55.613 (19.60)	57.155 (18.63)	57.273 (18.54)	< 0.001 [b]
Age Distribution						
18 – < 25	28 (5.2%)	3 (1.4%)	4 (6.5%)	123 (4.0%)	158 (4.1%)	< 0.001
25 – < 35	70 (13.1%)	4 (1.9%)	8 (12.9%)	305 (9.9%)	387 (9.9%)	
35 – < 45	68 (12.7%)	6 (2.9%)	9 (14.5%)	424 (13.7%)	507 (13.0%)	
45 – < 55	72 (13.5%)	33 (15.8%)	7 (11.3%)	515 (16.6%)	627 (16.1%)	
55 – < 65	102 (19.1%)	44 (21.1%)	10 (16.1%)	564 (18.2%)	720 (18.5%)	
65 – < 75	120 (22.4%)	45 (21.5%)	11 (17.7%)	540 (17.4%)	716 (18.4%)	
75 – < 85	52 (9.7%)	54 (25.8%)	10 (16.1%)	381 (12.3%)	497 (12.7%)	
85 – < 90	13 (2.4%)	14 (6.7%)	2 (3.2%)	143 (4.6%)	172 (4.4%)	
90+	10 (1.9%)	6 (2.9%)	1 (1.6%)	100 (3.2%)	117 (3.0%)	
Biological sex assigned at birth						
Male	260 (48.6%)	120 (57.4%)	29 (46.8%)	1,556 (50.3%)	1,965 (50.4%)	0.350
Female	275 (51.4%)	89 (42.6%)	33 (53.2%)	1,536 (49.6%)	1,933 (49.6%)	
Unknown	0 (0.0%)	0 (0.0%)	0 (0.0%)	< 10	< 10	
Ethnicity						
Not Hispanic	534 (99.8%)	207 (99.0%)	26 (43.3%)	2,231 (75.6%)	2,998 (79.9%)	< 0.001
Hispanic	1 (0.2%)	2 (1.0%)	34 (56.7%)	526 (17.8%)	563 (15.0%)	
Unknown	0 (0.0%)	0 (0.0%)	0 (0.0%)	193 (6.5%)	193 (5.1%)	
Highest level of education completed						
Did not complete Secondary School or less than High School	246 (46.0%)	< 10	15 (24.2%)	< 10	270 (6.9%)	< 0.001
Some Secondary School up to at least Associate’s or Technical Degree complete	62 (11.6)	< 10	25 (40.3%)	56 (1.8%)	145 (3.7%)	
At least College up to Postgraduate	87 (16.3%)	36 (17.2%)	19 (30.6%)	68 (2.2%)	210 (5.4%)	
Unknown	140 (26.2%)	164 (80.4%)	< 10	932 (30.1%)	1,237 (31.7%)	
*Missing*	*0 (0.0%)*	*< 10*	*< 10*	*2032 (65.6%)*	*2039 (52.3%)*	

[a] P-values were derived from ANOVA, Chi-Square or Fisher Exact Tests, when appropriate.

[b] Significant pairwise differences: Africa vs Asia, Africa vs US, Asia vs Europe, US vs Asia

**Table 3:** Demographic Characteristic by Income Group

Characteristics	Income Group			p-value [a]
	Low-Middle Income	High Income	Total	
N	744 (19.1%)	3,157 (80.9%)	3,901 (100.0%)	
age (Mean (SD)	57.902 (18.112)	57.124 (18.644)	57.273 (18.544)	0.005
Age Group				
18 – < 25	31 (4.2%)	127 (4.0%)	158 (4.1%)	< 0.001
25 – < 35	74 (9.9%)	313 (9.9%)	387 (9.9%)	
35 – < 45	74 (9.9%)	433 (13.7%)	507 (13.0%)	
45 – < 55	105 (14.1%)	522 (16.5%)	627 (16.1%)	
55 – < 65	146 (19.6%)	574 (18.2%)	720 (18.5%)	
65 – < 75	165 (22.2%)	551 (17.5%)	716 (18.4%)	
75 – < 85	106 (14.2%)	391 (12.4%)	497 (12.7%)	
85 – < 90	27 (3.6%)	145 (4.6%)	172 (4.4%)	
90+	16 (2.2%)	101 (3.2%)	117 (3.0%)	
Patient’s biological sex (sex assigned at birth)				
Male	380 (51.1%)	1,585 (50.2%)	1,965 (50.4%)	0.647
Female	364 (48.9%)	1,569 (49.7%)	1,933 (49.6%)	
Unknown	0 (0.0%)	3 (0.1%)	3 (0.1%)	
ethnicity				
Not Hispanic	741 (99.6%)	2,257 (75.0%)	2,998 (79.9%)	< 0.001
Hispanic	3 (0.4%)	560 (18.6%)	563 (15.0%)	
Unknown	0 (0.0%)	193 (6.4%)	193 (5.1%)	
Highest level of education completed				
Did not complete Secondary School or less than High School	248 (33.3%)	22 (0.7%)	270 (6.9%)	< 0.001
Some Secondary School up to at least Associate’s or Technical Degree complete	64 (8.6%)	81 (2.6%)	145 (3.7%)	
At least College up to Postgraduate	123 (16.5%)	87 (2.8%)	210 (5.4%)	
Unknown	304 (40.9%)	933 (29.6%)	1,237 (31.7%)	
*Missing*	*< 10*	*2034 (64.4%)*	*2039 (52.3%)*	

[a] P-values were derived from independent T-test, Chi-Square or Fisher Exact Tests, when appropriate.

**Table 4:** Clinical Characteristics per Region

Characteristics	Region Group					p-value [a]
	Africa	Asia	Europe	US	Total	
Neurologic Comorbidity Status	*535 (13.7%)*	*209 (5.4%)*	*62 (1.6%)*	*3,095 (79.3%)*	*3,901 (100.0%)*	
With	47 (8.8%)	73 (34.9%)	30 (48.4%)	1892 (61.1%)	2042 (52.3%)	< 0.001
Without	417 (77.9%)	127 (60.8%)	31 (50.0%)	746 (24.1%)	1321 (33.9%)	
Unknown	71 (13.3%)	9 (4.3%)	1 (1.6%)	457 (14.8%)	538 (13.8%)	
Specific Neurologic Comorbids (Top 5)	*47 (2.3%)*	*73 (3.6%)*	*30 (1.5%)*	*1892 (92.6%)*	*2042 (100.0%)*	
Comorbid Psychiatric Condition (e.g. mood or thought disorder)	< 10	< 10	13 (43.3%)	472 (24.9%)	493 (24.1%)	< 0.001
Comorbid Stroke (ischemic stroke, TIA, intracerebral hemorrhage, subarachnoid he	19 (40.4%)	42 (57.5%)	1 (3.3%)	263 (13.9%)	325 (15.9%)	< 0.001
Comorbid Headache	< 10	< 10	< 10	228 (12.1%)	234 (11.5%)	< 0.001
Comorbid Seizure Disorder or Epilepsy	< 10	< 10	< 10	225 (11.9%)	232 (11.4%)	< 0.001
Comorbid Dementia or Neurodegenerative Disease (Parkinson’s disease, Alzheimer’s	< 10	17 (23.3%)	< 10	208 (11.0%)	229 (11.2%)	< 0.001
Non-neurologic Comorbidity Status	*535 (13.7%)*	*209 (5.4%)*	*62 (1.6%)*	*3,095 (79.3%)*	*3,901 (100.0%)*	
With	319 (59.6%)	191 (91.4%)	46 (74.2%)	2399 (77.5%)	2955 (75.7%)	< 0.001
Without	171 (32.0%)	18 (8.6%)	16 (25.8%)	368 (11.9%)	573 (14.7%)	
Unknown	45 (8.4%)	< 10	< 10	328 (10.6%)	373 (9.6%)	
Specific Non-Neurologic Comorbids	*319 (10.8%)*	*191 (6.5%)*	*46 (1.6%)*	*2399 (81.2%)*	*2955 (100.0%)*	
Hypertension/high blood pressure-with or without treatment	196 (61.4%)	145 (75.9%)	29 (63.0%)	1,207 (50.3%)	1,577 (53.4%)	< 0.001
Diabetes	152 (47.6%)	109 (57.1%)	< 10	687 (28.6%)	957 (32.4%)	< 0.001
Hyperlipidemia	< 10	51 (26.7%)	13 (28.3%)	727 (30.3%)	799 (27.0%)	< 0.001
Obesity	< 10	23 (12.0%)	10 (21.7%)	243 (10.1%)	280 (9.5%)	< 0.001
Asthma	< 10	< 10	< 10	210 (8.8%)	230 (7.8%)	< 0.001
WHO Clinical Progression Score						
Ambulatory mild disease	130 (24.6%)	13 (6.2%)	24 (38.7%)	739 (46.3%)	906 (37.8%)	< 0.001
Hospitalized: moderate disease	254 (48.0%)	71 (34.0%)	29 (46.8%)	415 (26.0%)	769 (32.1%)	
Hospitalized: severe disease	85 (16.1%)	52 (24.9%)	< 10	208 (13.0%)	346 (14.4%)	
Dead	60 (11.3%)	73 (34.9%)	< 10	234 (14.7%)	375 (15.7%)	

[a] P-values were derived from ANOVA, Chi-Square or Fisher Exact Tests, when appropriate.

[b] Significant pairwise differences: Africa vs Asia, Africa vs US, Asia vs Europe, US vs Asia

WHO: World Health Organization

**Table 5:** Clinical Characteristics per Income Group

Characteristics	Income Group			p-value [a]
	Low-Middle Income	High Income	Total	
Neurologic Comorbidity Status				
With	120 (16.1%)	1922 (60.9%)	2042 (52.3%)	< 0.001
Without	544 (73.1%)	777 (24.6%)	1321 (33.9%)	
Unknown	80 (10.8%)	458 (14.5%)	538 (13.8%)	
Specific Neurologic Comorbids (Top 5)	*120 (5.9%)*	*1922 (94.1%)*	*2042 (100%)*	
Comorbid Psychiatric Condition (e.g. mood or thought disorder)	< 10	485 (25.2%)	493 (24.1%)	< 0.001
Comorbid Stroke (ischemic stroke, TIA, intracerebral hemorrhage, subarachnoid he	61 (50.8%)	264 (13.7%)	325 (15.9%)	0.885
Comorbid Headache	< 10	232 (12.1%)	234 (11.5%)	< 0.001
Comorbid Seizure Disorder or Epilepsy	< 10	226 (11.8%)	232 (11.4%)	< 0.001
Comorbid Dementia or Neurodegenerative Disease (Parkinson’s disease, Alzheimer’s	19 (15.8%)	210 (10.9%)	229 (11.2%)	< 0.001
Non-neurologic Comorbidity Status	*510 (17.3%)*	*2445 (82.7%)*	*2955 (100%)*	
With	510 (68.5%)	2445 (77.4%)	2955 (75.7%)	< 0.001
Without	189 (25.4%)	384 (12.2%)	573 (14.7%)	
Unknown	45 (6.0%)	328 (10.4%)	373 (9.6%)	
Specific Non-Neurologic Comorbids				
Hypertension/high blood pressure-with or without treatment	341 (66.9)	1,236 (50.6)	1,577 (53.4)	< 0.001
Diabetes	261 (51.2)	696 (28.5)	957 (32.4)	< 0.001
Hyperlipidemia	59 (11.6)	740 (30.3)	799 (27.0)	< 0.001
Obesity	27 (5.3)	253 (10.3)	280 (9.5)	< 0.001
Asthma	12 (2.4)	218 (8.9)	230 (7.8)	< 0.001
WHO Clinical Progression Score				
Ambulatory mild disease	143 (19.4%)	763 (46.0%)	906 (37.8%)	< 0.001
Hospitalized: moderate disease	325 (44.0%)	444 (26.8%)	769 (32.1%)	
Hospitalized: severe disease	137 (18.6%)	209 (12.6%)	346 (14.4%)	
Dead	133 (18.0%)	242 (14.6%)	375 (15.7%)	

[a] P-values were derived from independent T-test, Chi-Square or Fisher Exact Tests, when appropriate.

WHO: World Health Organization

**Table 6:** Health system-related profile per region

Characteristics	Region Group					p-value [a]
	Africa	Asia	Europe	US	Total	
N	535 (13.7%)	209 (5.4%)	62 (1.6%)	3,095 (79.3%)	3,901 (100.0%)	
Insurance						
Insured	535 (100.0%)	208 (99.5%)	61 (98.4%)	3,027 (97.8%)	3,831 (98.2%)	< 0.001
Uninsured	0 (0.0%)	< 10	< 10	68 (2.2%)	70 (1.8%)	
Received immunization for COVID-19						
No	264 (49.4%)	153 (73.2%)	5 (9.6%)	613 (28.7%)	1,035 (35.3%)	< 0.001
Yes, before COVID-19	87 (16.3%)	50 (23.9%)	14 (26.9%)	146 (6.8%)	297 (10.1%)	
Yes, after COVID-19	25 (4.7%)	< 10	33 (63.5%)	916 (42.8%)	976 (33.3%)	
Unknown	158 (29.6%)	< 10	0 (0.0%)	463 (21.7%)	625 (21.3%)	
Received COVID-19 medications						
No	28 (5.2%)	8 (3.8%)	5 (8.1%)	582 (24.8%)	623 (19.8%)	< 0.001
Yes	19 (3.6%)	201 (96.2%)	57 (91.9%)	1,572 (67.1%)	1,849 (58.7%)	
Unknown	487 (91.2%)	0 (0.0%)	0 (0.0%)	190 (8.1%)	677 (21.5%)	
Covid-19 Hospitalization						
No	122 (22.8%)	9 (4.3%)	23 (37.1%)	1,184 (38.3%)	1,338 (34.3%)	< 0.001
Yes, before COVID-19-associated illness	413 (77.2%)	200 (95.7%)	39 (62.9%)	1,751 (56.6%)	2,403 (61.6%)	
Unknown	0 (0.0%)	0 (0.0%)	0 (0.0%)	160 (5.2%)	160 (4.1%)	
Hospital Days	*359 (15.8%)*	*200 (8.8%)*	*37 (1.6%)*	*1,671 (73.7%)*	*2,267 (100.0%)*	
Hospital: Length of Stay (Mean (SD))	9.259 (8.498)	22.795 (27.531)	16.351 (18.016)	15.315 (19.658)	15.033 (19.458)	< 0.001 [b]
ICU Admission	*413 (17.2%)*	*200 (8.3%)*	*39 (1.6%)*	*1,751 (72.9%)*	*2,403 (100.0%)*	
No	163 (39.8%)	97 (49.0%)	29 (74.4%)	391 (23.7%)	680 (29.6%)	< 0.001
Yes, before COVID-19-associated illness	169 (41.2%)	101 (51.0%)	10 (25.6%)	1,196 (72.5%)	1,476 (64.3%)	
Unknown	78 (19.0%)	0 (0.0%)	0 (0.0%)	63 (3.8%)	141 (6.1%)	
ICU Days	*86 (16.0%)*	*100 (18.7%)*	*4 (0.7%)*	*346 (64.6%)*	*536 (100.0%)*	< 0.001 [c]
ICU: Length of Stay (Mean (SD))	7.674 (9.910)	18.070 (21.997)	10.500 (11.733)	13.965 (17.093)	13.696 (17.432)	
Total Death						
No	386 (72.1%)	88 (42.1%)	53 (85.5%)	1,423 (46.0%)	1,950 (50.0%)	< 0.001
Yes	81 (15.1%)	75 (35.9%)	< 10	906 (29.3%)	1,070 (27.4%)	
Unknown	68 (12.7%)	46 (22.0%)	< 10	766 (24.7%)	881 (22.6%)	

[a] P-values were derived from ANOVA, Chi-Square or Fisher Exact Tests, when appropriate.

[b] Significant pairwise differences: Africa vs Asia, Africa vs US, Asia vs Europe, US vs Asia

[c] Significant pairwise differences: Africa vs Asia, Africa vs US, Asia vs Europe

ICU: Intensive Care Unit

**Table 7:** Health System Profile per Income Group

Characteristics	Income Group			p-value [a]
	Middle Income	High Income	Total	
N	744 (19.1%)	3,157 (80.9%)	3,901 (100.0%)	
Insurance				
Insured	743 (99.9%)	3,088 (97.8%)	3,831 (98.2%)	< 0.001
Uninsured	1 (0.1%)	69 (2.2%)	70 (1.8%)	
Received immunization for COVID-19				
No	417 (56.1%)	618 (28.2%)	1,035 (35.3%)	< 0.001
Yes, before COVID-19	137 (18.4%)	160 (7.3%)	297 (10.1%)	
Yes, after COVID-19	27 (3.6%)	949 (43.3%)	976 (33.3%)	
Unknown	162 (21.8%)	463 (21.1%)	625 (21.3%)	
Received COVID-19 medications				
No	36 (4.8%)	587 (24.4%)	623 (19.8%)	< 0.001
Yes	220 (29.6%)	1,629 (67.7%)	1,849 (58.7%)	
Unknown	487 (65.5%)	190 (7.9%)	677 (21.5%)	
COVID-19 Hospitalization				
No	131 (17.6%)	1,207 (38.2%)	1,338 (34.3%)	< 0.001
Yes, before COVID-19-associated illness	613 (82.4%)	1,790 (56.7%)	2,403 (61.6%)	
Unknown	0 (0.0%)	160 (5.1%)	160 (4.1%)	
Hospital Days (if hospitalized)	*559 (24.7%)*	*1,708 (75.3%)*	*2,267 (100.0%)*	
Hospital: Length of Stay (Mean (SD))	14.102 (18.943)	15.337 (19.620)	15.033 (19.458)	0.193
ICU Admission (if hospitalized)	*613 (25.5%)*	*1,790 (74.5%)*	*2,403 (100.0%)*	
No	260 (42.8%)	420 (24.9%)	680 (29.6%)	< 0.001
Yes, before COVID-19-associated illness	270 (44.4%)	1,206 (71.4%)	1,476 (64.3%)	
Unknown	78 (12.8%)	63 (3.7%)	141 (6.1%)	
ICU Days (if hospitalized and admitted in ICU)	*186 (34.7%)*	*350 (65.3%)*	*536 (100.0%)*	0.676
ICU: Length of Stay (Mean (SD))	13.263 (18.195)	13.926 (17.034)	13.696 (17.432)	
Total Death				
No	474 (63.7%)	1,476 (46.8%)	1,950 (50.0%)	< 0.001
Yes	156 (21.0%)	914 (29.0%)	1,070 (27.4%)	
Unknown	114 (15.3%)	767 (24.3%)	881 (22.6%)	

[a] P-values were derived from ANOVA, Chi-Square or Fisher Exact Tests, when appropriate.

ICU: Intensive Care Unit

**Table 8:** Ranking of Neuro-COVID per Region Group

Top Conditions*	Total Sample (n = 3901)		US (n = 3095)		Europe (n = 62)		Asia (n = 209)		Africa (n = 535)	
	Rank	Count (%)	Rank	Count (%)	Rank	Count (%)	Rank	Count (%)	Rank	Count (%)
Neurocognitive Disorders	1	1491 (38.22)	1	1433 (46.30)	2	27 (43.55)	5	26 (12.44)	N/A	N/A
Fatigue disorders	2	1210 (31.02)	3	844 (27.27)	1	39 (62.90)	4	28 (13.40)	1	299 (55.89)
Headache	3	1096 (28.10)	2	990 (31.99)	N/A	N/A	3	33 (15.79)	5	55 (10.28)
Anosmia	4	891 (22.84)	4	825 (26.66)	3	22 (35.48)	N/A	N/A	N/A	N/A
Ageusia	5	863 (22.12)	5	808 (26.11)	5	19 (30.65)	N/A	N/A	N/A	N/A
Mood	N/A	N/A	N/A	N/A	4	20 (32.26)	N/A	N/A	2	224 (41.87)
Stroke	N/A	N/A	N/A	N/A	N/A	N/A	1	73 (34.93)	4	144 (26.92)
Seizures	N/A	N/A	N/A	N/A	N/A	N/A	2	39 (18.66)	N/A	N/A
Thought Disorder	N/A	N/A	N/A	N/A	N/A	N/A	N/A	N/A	3	151 (28.22)

* Reported as (1) new, (2) worsened, or (3) present, with unclear past history or unknown whether new or worsened

**Table 9:** Ranking of Neuro-COVID cases per income group

Top Conditions*	Total Sample (n = 3901)		High Income (n = 3157)		Middle Income (n = 744)	
	Top	Count (%)	Top	Count (%)	Top	Count (%)
Neurocognitive Disorders	1	1491 (38.22)	1	1460 (46.25)	N/A	N/A
Fatigue disorders	2	1210 (31.02)	3	883 (27.97)	1	327 (43.95)
Headache	3	1096 (28.10)	2	1008 (31.93)	5	88 (11.83)
Anosmia	4	891 (22.84)	4	847 (26.83)	N/A	N/A
Ageusia	5	863 (22.12)	5	827 (26.20)	N/A	N/A
Mood	N/A	N/A	N/A	N/A	2	229 (30.78)
Stroke	N/A	N/A	N/A	N/A	3	217 (29.17)
Thought Disorder	N/A	N/A	N/A	N/A	4	158 (21.24)

* Reported as (1) new, (2) worsened, or (3) present, with unclear past history or unknown whether new or worsened

**Table 10:** Top Neurologic Long COVID Symptoms

Condition [a]	With Timing Response [b]	Timing: >3 months [c]	Status	
			New	Worsened/Present but unclear timing or status
Fatigue	692	67 (9.7%)	66 (98.5%)	< 10
Mood	741	66 (8.9%)	61 (92.4%)	< 10
Thought Disorder	184	43 (23.4%)	42 (97.7%)	< 10
Stroke	526	29 (5.5%)	13 (44.8%)	16 (55.2%)
Neurocognitive Disorder	1348	23 (1.7%)	15 (65.2%)	< 10
Headache	912	17 (1.9%)	11 (64.7%)	< 10

[a] All other conditions had less than 10 responses for timing > 3 months

[b] Responses can be: < 1 month, 1 – < 3 months, >3 months, Unknown

[c] Subset of those with timing response; arranged by raw counts

## Data Availability

Data supporting this study will be available for sharing according to the NIH Data Management and Sharing Policy by contacting the COVID-19 Neuro Databank at NYU Langone Health.
